# Acoustic notch filtering earmuff utilizing Helmholtz resonator arrays

**DOI:** 10.1371/journal.pone.0258842

**Published:** 2021-10-19

**Authors:** Fumiya Mizukoshi, Hidetoshi Takahashi

**Affiliations:** Department of Mechanical Engineering, Faculty of Science and Technology, Keio University, Yokohama, Kanagawa, Japan; Universiti Brunei Darussalam, BRUNEI DARUSSALAM

## Abstract

In recent years, noisy bustling environments have created situations in which earmuffs must soundproof only specific noise while transmitting significant sounds, such as voices, for work safety and efficiency. Two sound insulation technologies have been utilized: passive noise control (PNC) and active noise control (ANC). However, PNC is incapable of insulating selective frequencies of noise, and ANC is limited to low-frequency sounds. Thus, it has been difficult for traditional earmuffs to cancel out only high-frequency noise that people feel uncomfortable hearing. Here, we propose an acoustic notch filtering earmuff utilizing Helmholtz resonator (HR) arrays that provides a sound attenuation effect around the tuneable resonant frequency. A sheet-like sound insulating plate comprising HR arrays is realized in a honeycomb structure. Since the resonant frequency is determined by the geometry of the HR arrays, a highly audible sound region can be designed as the target frequency. In this research, the acoustic notch filtering performance of the proposed HR array plate is investigated in both simulations and experiments. Furthermore, the fabricated earmuffs using the novel HR array plates achieve a sound insulation performance exceeding 40 dB at the target frequency, which is sufficiently high compared to that of conventional earmuffs. The experimental results confirm that the proposed device is a useful approach for insulating frequency-selective sound.

## Introduction

Acoustic noise is one of the most serious environmental problems due to its variety of negative effects on people’s health and lives; for example, acoustic noise is responsible for causing sleep disturbances, hearing impairment, and heart disease [1–3]. To protect ears from acoustic noise and provide a comfortable acoustic environment, various sound insulation technologies have been developed; these technologies are mainly classified as either passive noise control (PNC) [4–10] or active noise control (ANC) [11–23]. PNC is based on physical sound insulation using a sound insulating material without a power supply, whereas ANC attenuates acoustic noise by generating waves of the opposite phase to the noise (i.e., anti-noise) from a loudspeaker.

Two forms of sound insulation technologies are generally applied: wide walls (such as sound barriers) and small devices (such as those used for hearing protection). In the former, PNC is commonly used because a large area has to be uniformly and permanently soundproofed. Over the past couple of decades, as PNC performance has evolved, several studies have presented improved sound insulation technologies via multiple materials [10], and sound insulation with perforated boards has been developed to ensure atmospheric permeability [9]. In contrast, it is not easy to apply ANC to sound insulation over a wide area because of the sound field. Hence, ANC has been employed for applications in confined spaces, such as car interiors [20, 21] and windows [17], using multiple microphones and loudspeakers. In the latter, such as wearable devices, two methods are widely used: PNC alone and a combination of PNC and ANC. Earplugs and earmuffs are traditional sound insulation devices that utilize only PNC [4]. However, since PNC cannot select the frequency of sound insulation and does not provide sufficient sound insulation for low-frequency noise in principle, the combination of PNC and ANC was proposed [12, 16, 18]. Improvements in the algorithm have increased the computational efficiency, providing a response to fast changes in signal characteristics [11, 13–15, 23]. Furthermore, recent studies have shown that subband ANC and frequency-domain constraint algorithms can solely cancel noise at specified frequencies [13, 15, 23]. However, in principle, ANC still has an upper limit to its working frequency. In earmuff-type sound insulation devices, ANC is generally effective at frequencies below 1.5 kHz, when the wavelength is sufficiently longer than the dimensions of the surrounding space. At these frequencies, ANC becomes dominant over PNC. Thus, with ANC for low-frequency noise and PNC for high-frequency noise, noise can be insulated over a wide frequency range.

Earmuff-type devices with PNC and ANC are widely marketed as “noise-cancelling headphones”, which are useful only when the headphones cover the ears. However, these headphones are not suitable for all situations as noise-cancelling earmuffs. For instance, shutting out important sounds (such as voices) at the same time as noise can reduce work safety and efficiency, especially in situations that require the exchange of important information through oral communication. In these situations, sound insulation devices should block noise at only specific noise frequencies. Furthermore, specific monotone noise at audible frequencies is often generated by machines, engines, animals, etc. [24, 25]. However, it is difficult for conventional PNC and ANC technologies to cut only specific high frequencies due to their frequency characteristics.

Here, we focus on the muffling effect of a Helmholtz resonator (HR) as an alternative to PNC and ANC. An HR composed of a chamber with a neck-shaped orifice (neck) has a muffling effect around the resonant frequency without a power supply. By varying the dimensions of the HR, the resonant frequency becomes selectable in the mid-high audible frequency range (1–20 kHz); consequently, the optimum sound insulation effect can be achieved according to the environment of use in the frequency range where ANC is not practical. Applying this effect, it is possible to insulate noise without excluding other important sounds. In this research, we propose an earmuff-type device by effectively employing an in-plane array of HRs that insulates only noise.

## Results

### Theory and design

**Fig 1:**
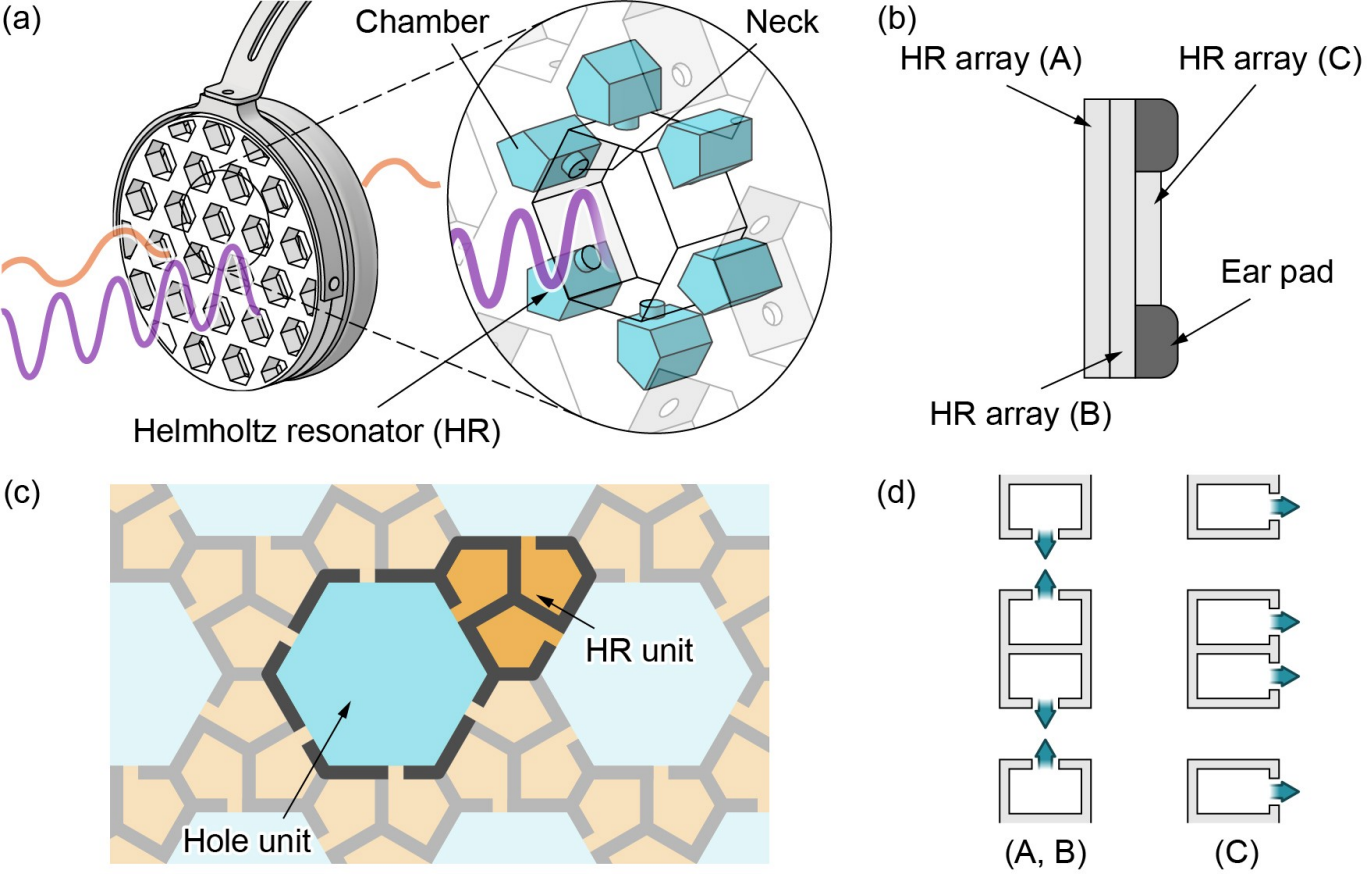
(a) Conceptual diagram of the device. Each HR structure is a chamber with a neck-shaped orifice (neck). Six parallel HRs are placed around each hole. The cancellation of waves from the HRs insulates noise at a specific frequency. (b) Cross-sectional view of the device. The device comprises an ear pad and three HR array plates for both the right and left ears. (c) Schematic diagram of the HR array unit structure. An HR array consists of regular hexagonal penetration hole units and hexagonal parallelogram HR units. An HR unit is an aggregation of three identical HRs. (d) Two HR array (A, B) plates insulate the sound waves entering perpendicularly to the plates, while the last HR array (C) plate insulates the sound waves entering parallel to the plates.

The HR is a famous acoustic phenomenon as an enhancement effect due to resonance [26]. As a counterpart to the enhancement effect, a muffling effect also appears that can be used for reducing noise. Here, multiple HR structures are density-arrayed in the plane for use as earmuffs. A schematic image of the proposed acoustic notch filtering earmuff is shown in Fig 1(A) and 1(B). The device consists of a headphone frame, an ear pad, and three HR array plate layers. We set the size and mass of the plates to be on the order of 200 mm in diameter and 500 g, respectively, which are sufficiently low for a person to comfortably wear. The HR array is a plane-filling structure consisting of HR units for attenuating noise and penetration hole units for transmitting sound. Considering that the noise insulation performance of a typical earmuff device is approximately 20–30 dB [27], we set the required specification to exceed 20 dB.

Conventionally, the muffling effect of a single HR has been used in long and narrow holes that can be regarded as one-dimensional sound fields, such as long ducts [28–35]. In general, if the axial length of the hole corresponding to the sound field is not sufficiently long compared to the wavelength, interference causes a pressure gradient in the radial direction of the hole. As a result, the HR muffling effect becomes insufficient. To solve this problem, our group previously reported that multiple HRs placed around a short hole can function as a planar acoustic notch filter [36]. A sufficient sound insulation effect was obtained over a short distance (less than the wavelength) via multiple HRs. Based on the effect of multiple HRs, in this research, short holes surrounded by multiple HRs are densely arrayed in the plane to insulate sound over a large area with a thin plate. Here, the HR array is based on a honeycomb structure to increase the strength and area efficiency. By increasing the area efficiency, i.e., increasing the number of holes and HRs per unit area, it is possible to improve the sound insulation effect and the permeability of other sounds while reducing the thickness and weight of the device. The HR array consists of two types of hexagonal units: regular hexagonal penetration hole units and hexagonal parallelogram HR units, where each HR unit is an aggregation of three identical HRs (Fig 1(C)). The hole units and HR units are densely arrayed at a 1:2 ratio; thus, six HRs are placed around each hole. These six HRs are connected via necks on the inner side of each hole so that cancelling waves from the HRs operate on the sound waves passing through each hole. The HR array structure is described in more detail in the Methods section.

The resonant frequency of an HR is calculated by the following equation:

$$f_{0}=\frac{c}{2\pi }\sqrt{\frac{S}{(l+\delta_{e}+\delta_{i})V}}$$ (1)

where *c* is the speed of sound in the medium (air), *V* is the chamber volume, *S* is the neck aperture area, and *l* is the neck length. *δ*_*e*_ and *δ*_*i*_ are the neck aperture correction due to the motion of gas particles outside the resonator and inside the resonator, respectively. When the HR is mounted onto a duct, the exterior aperture correction *δ*_*e*_ is affected by the relationship between the duct and neck pipe diameters [37]. Ji [30] suggested two curve-fitted expressions for neck-duct interface aperture correction for a circular duct-HR system;

$$\{\begin{array}{c} \frac{\delta_{e}}{r}=0.8216-0.0644(\frac{r}{a})-0.694{\left(\frac{r}{a}\right)}^{2},\frac{r}{a}\leq 0.4\\ \frac{\delta_{e}}{r}=0.9326-0.6196(\frac{r}{a}),\frac{r}{a}>0.4 \end{array}$$ (2)

where *r* is the radius of the neck of the HR and *a* is the radius of the duct.

The interior aperture correction *δ*_*i*_ is affected by the shape of the chamber. Ingard [38] and Alster [39] investigated the effects of the geometry on the resonance frequency of the HR. In this study, the HR has a complex polyhedron-shaped chamber. For simplicity, we assume that the shape of the chamber is a sphere with the same volume. Alster suggested an equation evaluating the interior aperture correction of a spherical HR:

$$\{\begin{array}{c} \delta_{i}=\frac{r^{2}}{R}\frac{1}{2-A}[\frac{1}{3}-\frac{1}{2(A+1)}-\frac{2}{{\left(A+1\right)}^{2}}+\frac{4}{{\left(A+1\right)}^{3}}\ln\frac{2}{1-A}]\\ A=\sqrt{1-{\left(\frac{r}{R}\right)}^{2}} \end{array}$$ (3)

where *R* is the radius of the chamber of the HR. The resonant frequency *f*_0_ is calculated by substituting Eqs (2) and (3) into Eq (1). An HR has both an enhancing effect and a muffling effect just before and after the resonant frequency, and we use these equations to calculate the notch frequency.

**Fig 2:**
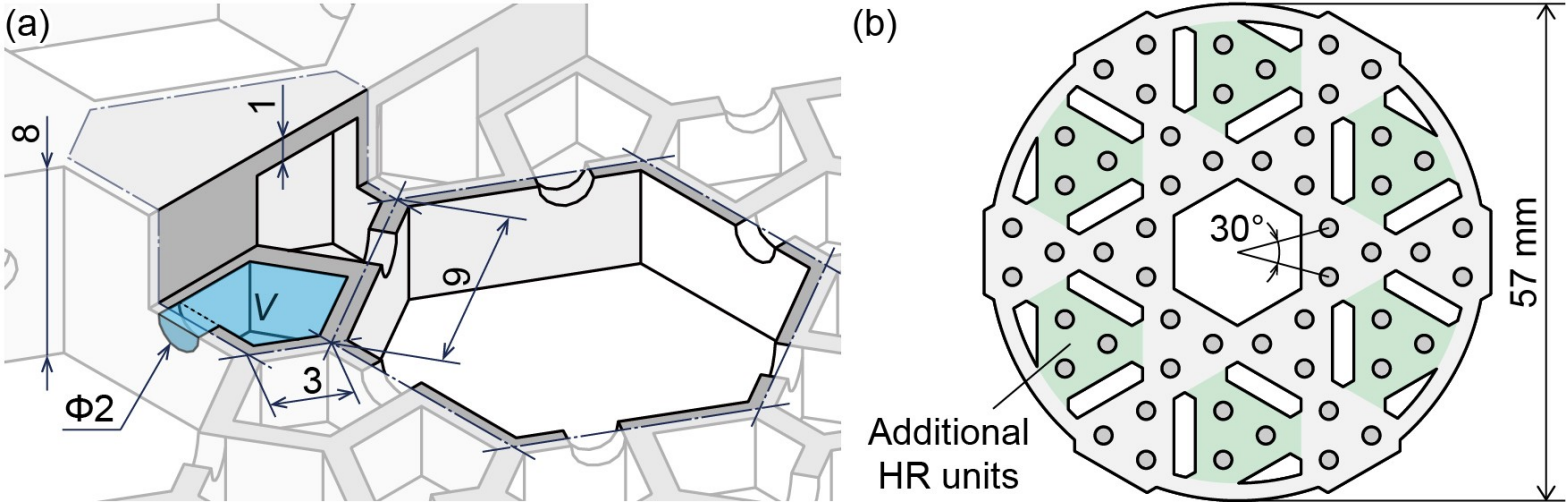
(a) Design of the HR units and penetration hole units, which are shaped like a hexagonal parallelogram and a regular hexagon, respectively. (b) Design of the HR array (C) plate. The necks are connected to the front surface of the plate.

As the cut-off frequency of the HR, we chose 6 kHz from within the audible range. This frequency is characterized by uncomfortable high-frequency noise that is difficult to cancel out by ANC headphones. Examples of such sounds include metallic sounds and noises produced by high-speed rotating objects or periodic magnetic fields. The dimensions of the chamber and neck were determined according to this frequency. The overall thickness of a single HR array was set to 8 mm, considering that it is intended to be integrated into the earmuffs. Considering the strength and weight of the device, all the internal partition walls were set to be 1 mm thick. An enlarged view of the units of the designed HR array is shown in Fig 2(A). The HR array is a plane-filling structure consisting of two types of hexagonal units: regular hexagonal units with a side length of 9 mm (hole units) and hexagonal parallelogram units with side lengths of either 3 mm or 9 mm (HR units). The HR array is designed from these unit structures arrayed on a plane. In this case, the aperture ratio of the HR array (area ratio between the penetration hole unit and HR unit) is 0.483 so that sounds except noise pass with minimal attenuation. Each HR unit is partitioned into three equal interior chambers, and each of these three interior HRs is connected to a different hole. The neck diameter is *r* = 2 mm, and the neck length is equal to the inner wall thickness (*l* = 1 mm). The volume of each chamber is *V* = 115 mm^3^, and the hypothetical radius of the chamber is calculated to be *R* = 115 mm, where *R* is a value that satisfies *V* = 4π*R*^3^/3. Here, we assume that the shape of the hole is a cylindrical duct with a radius of 8 mm. Then, the resonant frequency is calculated to be *f*_0_ = 5.98 kHz from Eqs (1), (2), and (3). From this HR array structure, we designed circular HR array plates with a diameter of 108 mm to match the shape of the ear pads to be integrated into an earmuff-type device, which we call an HR array earmuff hereinafter. The HR array plate has 27 hole units and 46 HR units.

We analysed the effect of viscous loss in this design. Viscous loss is more pronounced when the sound wave passes through a narrow tube and the resonators are in a state of resonance. The boundary layer thickness *d* is expressed as follows:

$$d=\sqrt{2\eta /\rho_{0}\omega }$$ (4)

where *η* is the dynamic viscosity, *ρ*_0_ is the equilibrium medium density, and *ω* is the angular frequency. When the dynamic viscosity and the density of air are *η* = 18.13 × 10^−6^ Pa·s and *ρ*_0_ = 1.166 kg/m^3^, respectively, *d* = 0. 0407 mm is calculated at the resonance frequency *f*_0_ = 5.98 kHz. This is diameter is sufficiently smaller than the diameter of the pipe at the neck, indicating that the effect of viscous loss is negligible.

**Fig 3:**
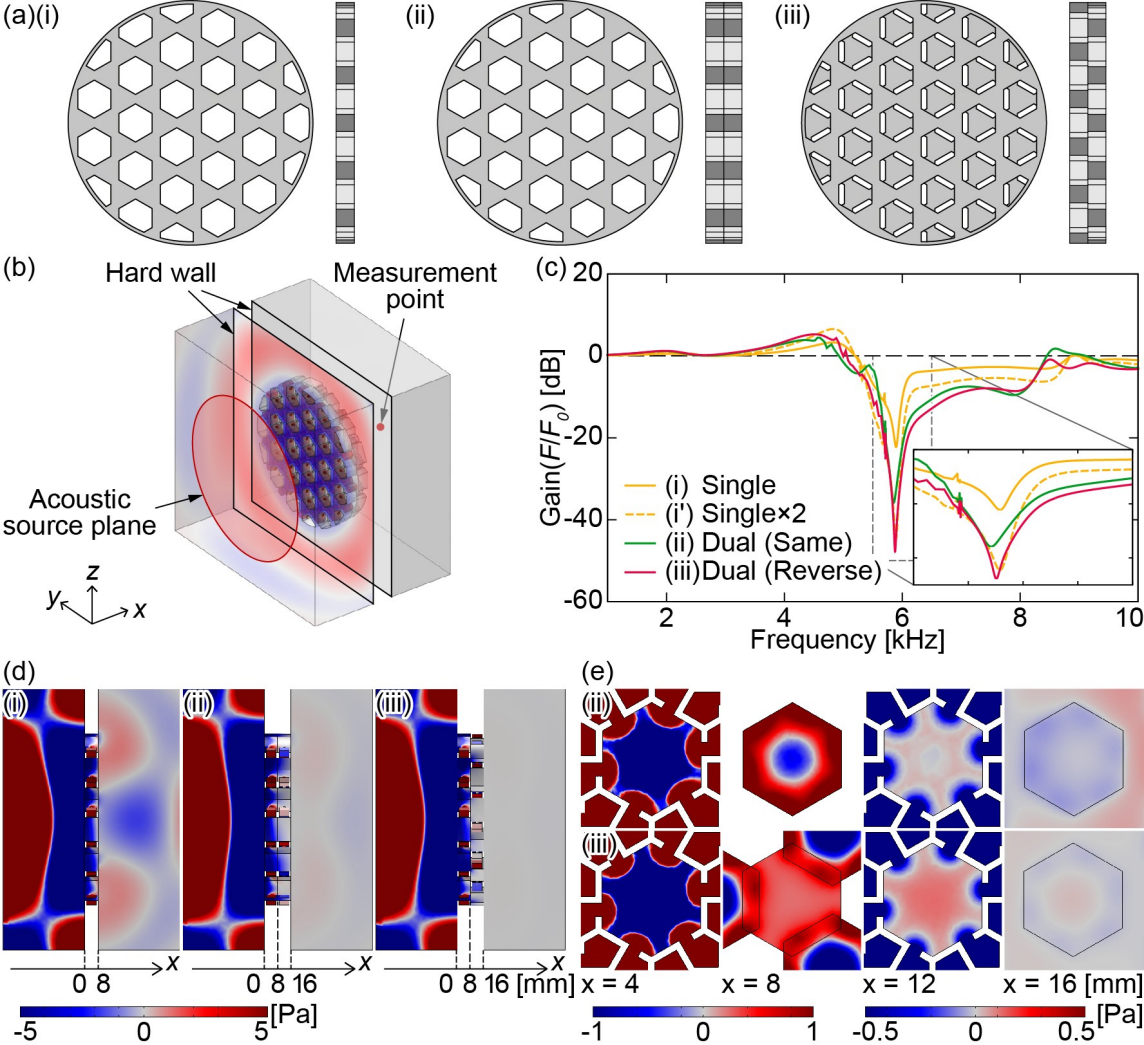
(a) Models of the single or combined HR array (A, B) plates. We investigated the sound insulation effect of these HR array plate models: (i) single, (ii) dual, same direction, and (iii) dual, opposite direction. (b) Simulation model. Sound waves are applied perpendicularly to the plates. (c) Simulated frequency responses of the HR array plates. (i’) Spectra of (i) doubled. (d) Sound pressure distributions viewed from the *z*-axis direction ((i) 5896 Hz, (ii) 5856 Hz, (iii) 5878 Hz). (e) Sound pressure distributions viewed from the *x*-axis direction enlarged about one hole ((ii) 5856 Hz, (iii) 5878 Hz).

Additionally, the sound insulation effect of the HR array plate can be enhanced by using multiple layers. In this study, two plates with the same units (HR array (A, B) plates) are installed on each side of the HR array earmuff (Fig 1(B)). We investigated two ways of layering the HR array plates: one where the holes of the two HR array plates are aligned and the other where the holes are offset (Fig 3(A)). The circular HR array plates were designed so that the positions of the penetration hole units and HR units are offset from each other when the two plates are placed in opposite directions. On the other hand, unlike the HR array structure, the spongy ear pads do not completely block sound waves, and thus, some acoustic waves can enter through the ear pads from the direction parallel to the plates. Therefore, an additional (third) HR array plate (HR array (C) plate) is installed inside the ear pad. The HR array (C) plate was designed with a diameter of 57 mm to match the inner diameter of the ear pad (Fig 2(B)). To match the notch frequencies of the HR array (A, B) plates, the unit structure is the same as that of the HR array (A, B) plates. The necks are connected to the front surface of the plate to insulate sound waves parallel to the plate (Fig 1(D)). In each HR unit, the three necks are located at the vertices of an equilateral triangle. Any pair of necks is positioned at a subtended angle of 30° from the centre of the adjacent unit. For the HR array (C) plate, six additional HR units were added to the original HR array (A, B) plates to improve the sound insulation performance; the additional HR units are shown in green in Fig 2(B). The narrow penetration holes between adjacent HR units provide pathways for vertically entering sound waves. To increase the transmittance of vertical sound waves, the centre of the HR array (C) plate is not equipped with HR units but is instead a large throughgoing hole. The HR units are arranged in a donut shape to effectively insulate sound waves entering perpendicularly from the sides of the plate. Even with three layered plates, the overall thickness is only 24 mm, which is sufficiently thin for earmuffs.

### Simulation 1: Vertical sound waves

To investigate the sound insulating effect of the HR array plates, an acoustic simulation was performed using finite element method (FEM) simulation software (COMSOL Multiphysics 5.5, COMSOL Inc., Sweden). As shown in Fig 3(A), three different models were compared: (i) a single plate, (ii) two plates in the same direction, and (iii) two plates installed in opposite directions. The sound waves are applied vertically to the plates, and the simulation does not consider the sound wrapping around from outside the plates. A sound pressure of 10 Pa is applied from a circular acoustic source plane located in the -*x* direction. The measurement point is positioned at the centre of the *yz* boundary plane located at a distance of 50 mm in the +*x* direction from the HR array plates (Fig 3(B)).

The size of the computational domain is 160 mm × 160 mm × (100 mm + *b*_*sum*_), where *b*_*sum*_ is the thickness of the HR array plate (*b*_*sum*_ = 8 mm for a single HR array plate and *b*_*sum*_ = 16 mm for two plates). The propagation of sound through the solid part is not considered in the simulation. The entire medium is composed of air. The state parameters of the air are set to a temperature of 293.15 K and an absolute pressure of 1 atm. A circular acoustic source plane with a diameter of 120 mm (pressure *p*_*a_out*_ = 10 Pa) is placed in the *yz* plane (-*x* direction). All other boundary surfaces are set to open conditions, and the impedance is set to match that of the air. The impedance *Z*_*j*_ is calculated as *Z*_*j*_ = 1.2 kg/m^3^ × 343 m/s from the density of the medium and the speed of sound. Either a single HR array plate or two HR array plates are placed at the centre of the *x*-axis direction of the computational domain. A hard wall is placed around the HR array plates to prevent sound from travelling around the plates. The centre of the boundary plane (*yz* plane, -*x* direction) is set as the measurement point. The element size parameters used to generate the finite element mesh are shown in Table 1 in S1 Appendix.

The frequency-domain analysis is performed in the range from 1 kHz to 10 kHz. In the range from 4 kHz to 6 kHz, the frequency step size is set to 1 Hz for a detailed investigation, whereas in the remainder of the frequency range, the frequency step size is set to 40 Hz. In addition to the three models shown in Fig 3(A), simulations are also performed for each model without HR units (i.e., models with only penetration holes). The frequency responses of the sound pressure level (SPL) with and without HR units are compared, and the difference, i.e., the gain (Gain(*F*/*F*_0_)), is calculated. Here, *F* and *F*_0_ denote the transfer functions between the source and the measurement point with and without HR units, respectively, corresponding to the experimental calculation described in the Experiment 1 subsection. Fig 1(A) and 1(B) in S1 Appendix shows the SPL frequency responses used to calculate the gain.

The calculated Gain(*F*/*F*_0_) values are shown in Fig 3(C). The notch frequency is approximately 5.9 kHz for all the models, which almost matches the calculated value of *f*_0_ = 5.84 kHz, although there is a variation of 40 Hz among the models. The sound insulation effect at each notch frequency becomes approximately 22 dB for a single plate, 36 dB for two plates (same direction), and 48 dB for two plates (opposite directions). High sound insulation effects are obtained near the notch frequency, confirming that the HR array plates function effectively as an acoustic notch filter.

Fig 3(D) shows the sound pressure distributions viewed from the *z*-axis direction at each notch frequency for the three models corresponding to Fig 3(A). The surface plane of the HR array plate closest to the source is defined as *x* = 0 mm, and the plane closest to the measurement point is *x* = 16 mm. The sound pressure from the source is attenuated by the HR array plates over the end surface of the plate (*x* = 16 mm). Compared to the single-plate model, the two-plate models more strongly attenuate sound waves. To closely investigate the sound insulation effect of the two-plate models, the sound pressure distributions on the *yz* plane (*x* = 4, 8, 12, 16 mm) for (ii) and (iii) are shown in Fig 3(E). The sound pressure distributions surrounding one hole in the centre of the HR array plate is magnified for clarity. The sound pressure is plotted in two dimensions in the range from -1 to 1 Pa for *x* = 4 and 8 mm and in the range from -0.5 to 0.5 Pa for *x* = 12 and 16 mm. In the sound pressure distributions for *x* = 4 and 12 mm, the sound waves entering the hole are clearly affected by the antiphase waves from the six surrounding HRs, causing a muffling effect in the hole. Hence, the sound pressure is sufficiently attenuated at the surface of the plate closest to the measurement point (*x* = 16 mm).

We further investigated the frequency response when the dimensions of each HR were distributed. The neck was shifted by two different factors, 1.1 and 1.05, and two different methods of distribution were investigated (Fig 2(A) in S1 Appendix). In model (i), the radii of all necks are equal. In model (ii), the neck radii are distributed within a single plate. In model (iii), while the radii of all necks in each plate are equal, each plate has different neck radii. Simulations were performed for five different models.

The simulated frequency responses are shown in Fig 2(C) and 2(D) in S1 Appendix. The spectra shown correspond to the case where the shift factor is set to 1.1 and 1.05, respectively. As shown, the larger the shift factor is, the wider the dip. Comparing models (ii) and (iii), (ii) shows an increase in the gain in the middle of the two notches. The notch value is flatter than that in model (i) with the same radius neck.

### Simulation 2: Parallel sound waves

**Fig 4:**
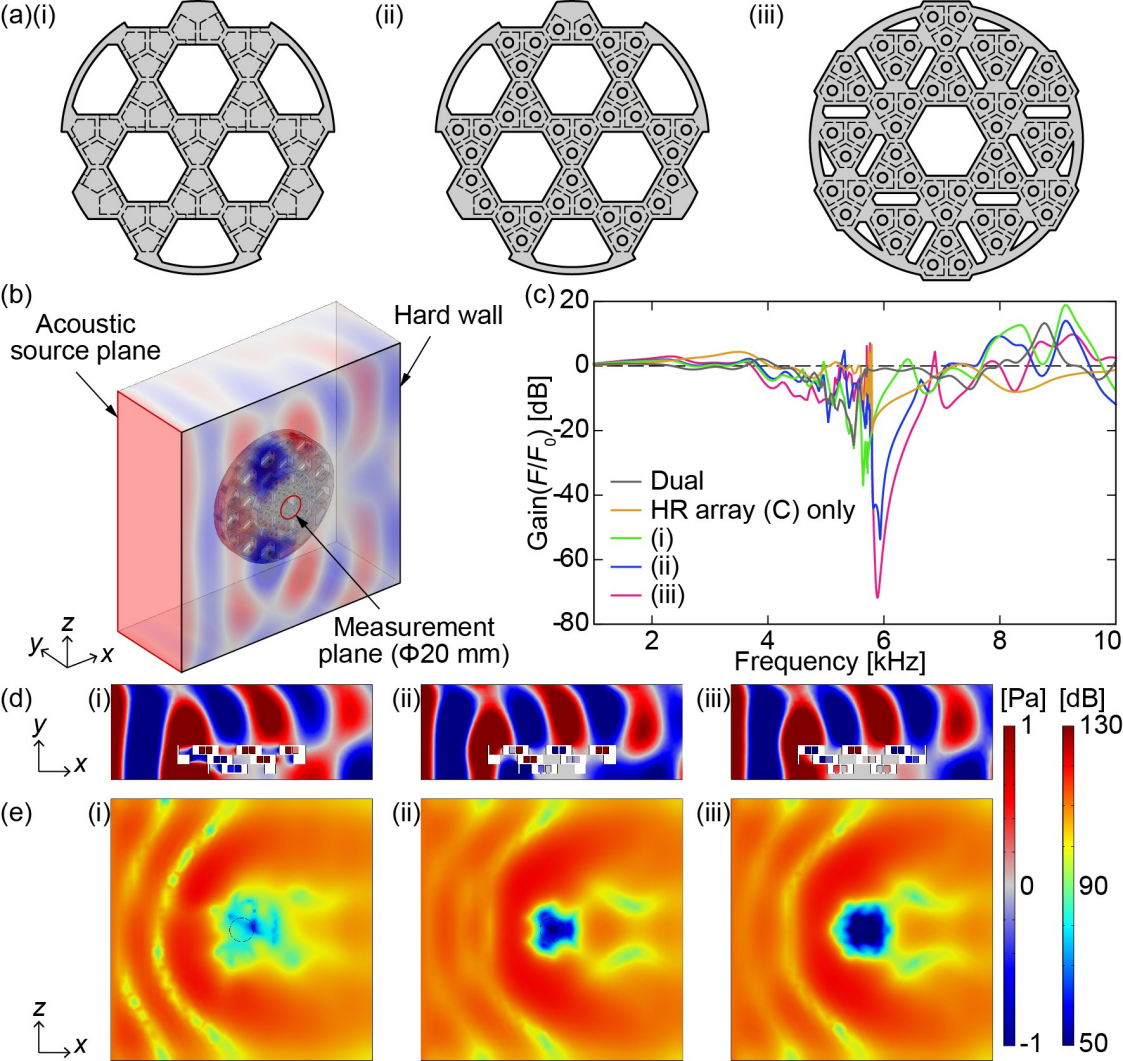
(a) HR array (C) plate models. (i) The necks are connected to the side of the penetrating hole. (ii) The necks are connected to the front surface of the plate. (iii) There are more HR units than in the model of (ii). (b) Simulation model. Sound waves are applied parallel to the plate. (c) Simulated frequency responses to parallel sound waves. (d) Sound pressure distributions viewed from the *z*-axis direction and (e) SPL distributions viewed from the *y*-axis direction ((i) 5640 Hz, (ii) 5935 Hz, (iii) 5889 Hz).

To confirm the sound insulation effect of the HR array (C) plate, an additional simulation was performed with sound waves applied parallel to the three HR array plates layers, namely, the HR array (A, B) plates and the HR array (C) plate. Here, we considered sound wrapping around from outside the plates. Three different models of the HR array (C) plate shown in Fig 4(A) were evaluated in terms of the sound insulation effect. Fig 4a-4i shows the model (i) with the necks connected to the side of the holes as well as the HR array (A, B) plates. Model (ii) in Fig 4(a-ii) has the same shape as model (i) from the front view; however, the necks are formed on the front surface of the plate. In model (iii) shown in Fig 4(a-iii), in addition to the necks being connected to the surface, the number of HR units is increased, and the area of the penetration holes is decreased. Here, the HR units are arranged in a donut shape for efficient sound insulation. The centre of the plate is designed as a large hole to improve the transmission of other sounds. Each of these three types of HR array (C) plates was analysed. In addition, we analysed a model without HR array (C) plates (a two-plate model with HR array (A, B) plates only) and a model with only an HR array (C) plate (model (iii)) for a total of five models.

The simulation model is shown in Fig 4(B). The size of the computational domain is 220 mm × 220 mm × 80 mm. Three HR array plates are placed parallel to the *yz* plane at a distance of 5 mm from the boundary surface. The *yz* boundary (+*x* direction) is set as a hard wall, while all other boundary surfaces are open. An acoustic source plane is placed on the *zx* boundary (-*y* direction). A circular area (20 mm diameter) in the centre of the *yz* boundary (+*x* direction) is employed as the measurement area.

The simulated frequency responses are shown in Fig 4(C). Similar to Simulation 1, the spectra were normalized with respect to the analysis results for the models without HR units. The model with only two HR array (A, B) plates and the model with only an HR array (C) plate do not have a sufficient sound insulation effect. On the other hand, the model with three HR array (A, B, C) plates has a high sound insulation effect around the notch frequency. Comparing the three models comprising three HR array (A, B, C) plates, the sound insulation effect is approximately 34 dB for model (i), approximately 56 dB for model (ii), and approximately 72 dB for model (iii) at each notch frequency. These results confirm that adding the HR array (C) plate to the HR array (A, B) plates improves the sound insulation effect for sound waves parallel to the plates. Fig 4(D) and 4(E) shows the sound pressure distribution in the *z*-axis direction and the SPL distribution in the *x*-axis direction at each notch frequency. The SPL evidently decreases at the centre of the plates; in particular, models (ii) and (iii) show a significant decrease at the centre. Comparing models (ii) and (iii) reveals that the range of the sound insulation effect can be expanded by arranging the HR units densely in a donut shape. Consequently, model (iii) has the highest sound insulation effect and the widest range of sound insulation effects.

### Experiment 1: Frequency response of HR array plates

**Fig 5:**
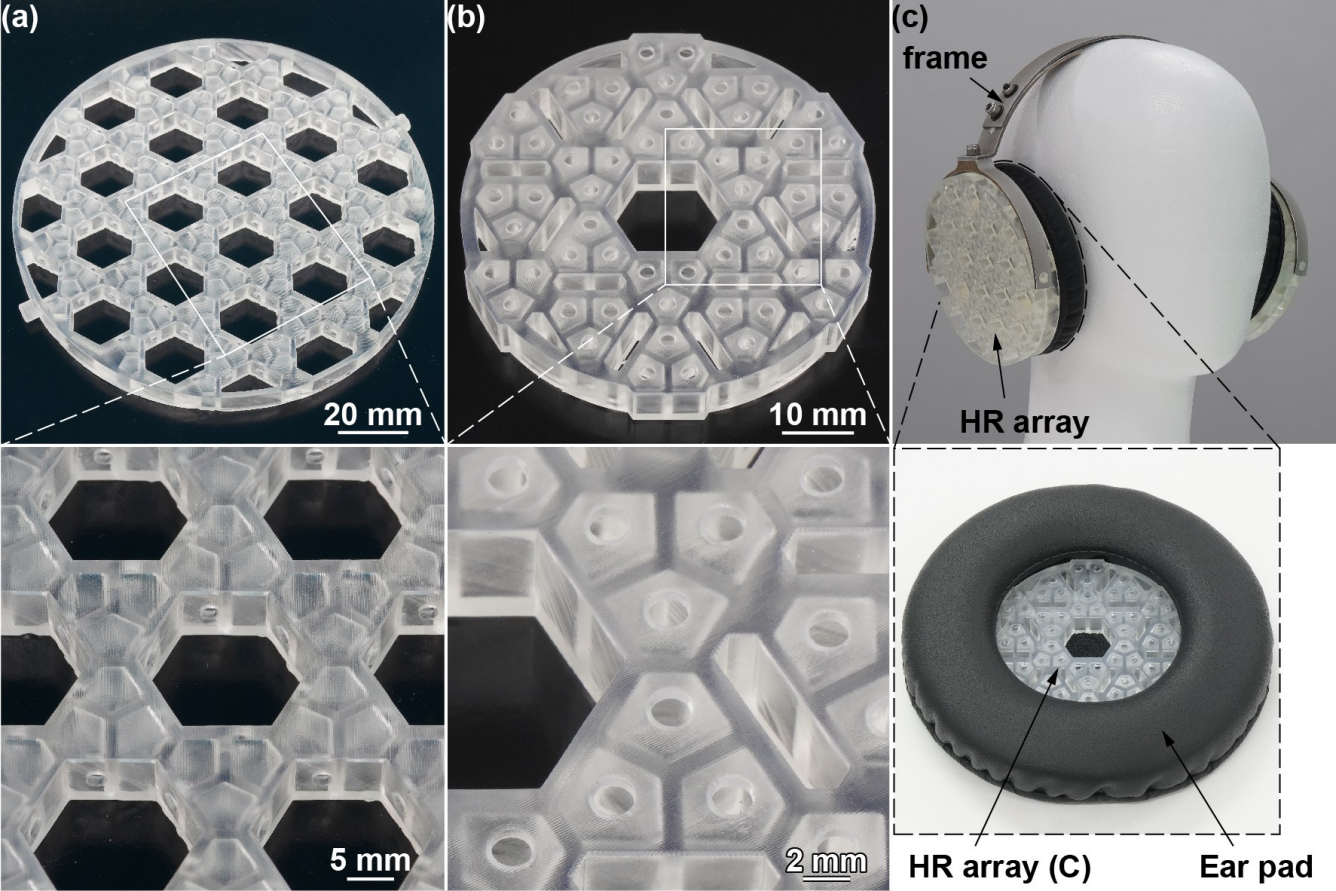
(a) HR array (A) plate fabricated using an SLA 3D printer. The chambers were moulded properly, and the necks were connected to the inner side of each penetration hole. (b) Fabricated HR array (C) plate. The necks were connected to the front surface of the HR array plate. (c) Fabricated HR array earmuff. Two HR array (A, B) plates were layered on the outside of each ear pad, while the last HR array (C) plate was inserted inside each ear pad.

The HR array (A, C) plates, fabricated using a stereolithography (SLA) 3D printer, are shown in Fig 5(A) and 5(B). Each chamber was correctly moulded with little resin residue inside the HR chamber. We installed the plates on a headphone frame to develop the device (the acoustic notch filtering earmuff) (Fig 5(C)). The aluminium frame is movable in three directions to fit the shape of the head. The HR array (C) plate is fitted inside the ear pad. The total weight of the device including all the plates on both ear sides is 266 g, which satisfies the required specifications.

**Fig 6:**
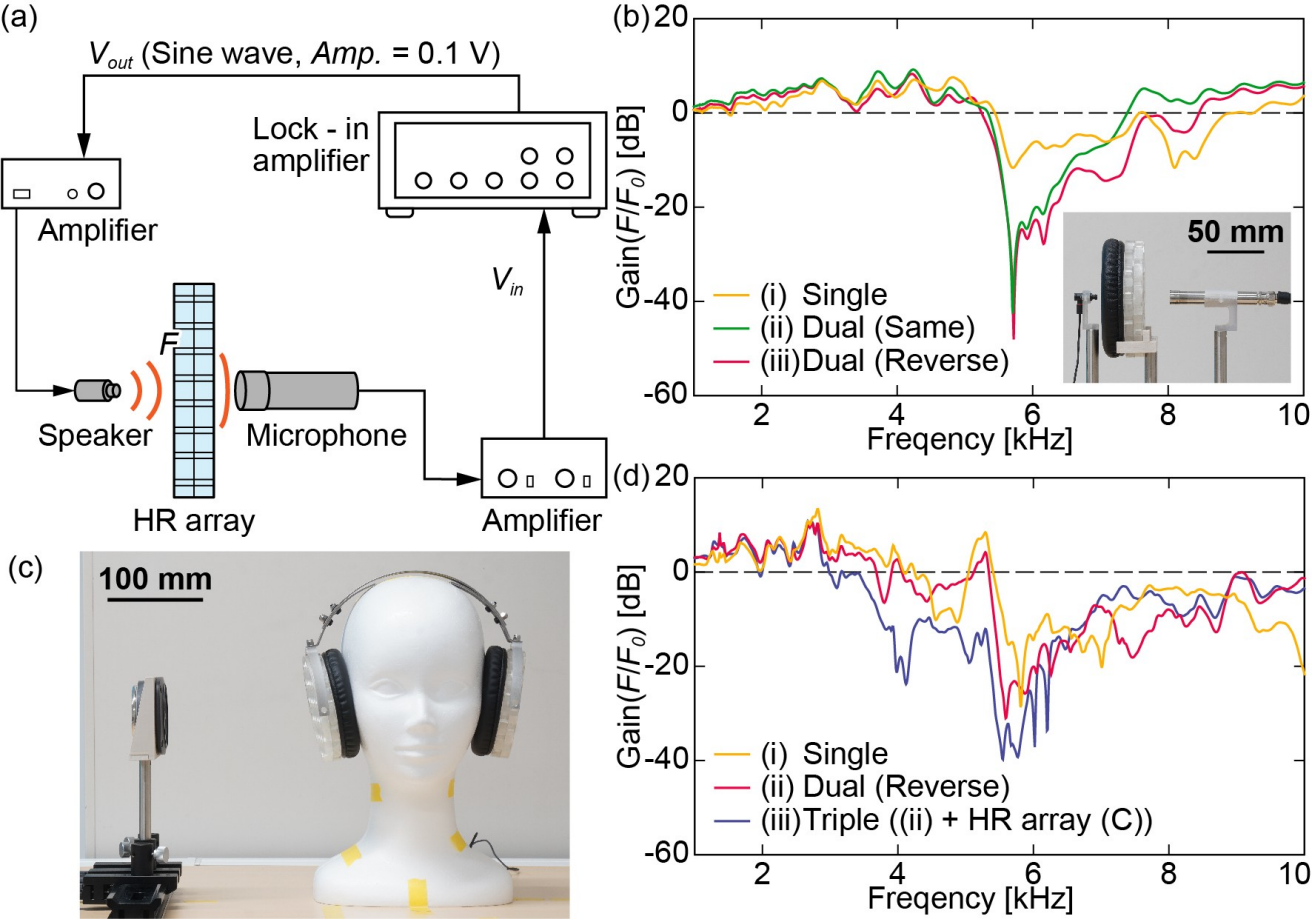
(a) Schematic image of the experimental setup. (b) Results of the experiment with a speaker, a microphone, and HR array plates. The graph shows the experimental frequency responses of the HR array plates. (c) Experimental setup with a speaker, the dummy head microphone, and the HR array earmuff. (d) Results of the experiment with the setup in (c). The graph shows the sound insulation performance of the device.

To evaluate the sound insulation effect, we conducted a sound experiment, as depicted in Fig 6(A). An earphone, the HR array plates, and a microphone were arranged along the same straight horizontal line. The sound waves emitted from the earphone reached the microphone through the HR array plates. As in the simulations, the frequency responses of the single plate, the two plates installed in the same direction, and the two plates installed in opposite directions were investigated. To eliminate the frequency characteristics of the experimental equipment from the result, the microphone signal without passing through the HR array plates was used as the reference.

The experimental procedure is described below. An earphone speaker (E4000, S’Next Co., Ltd., Japan), the fabricated HR array plates and a microphone (MI-3170, Ono Sokki Co., Ltd., Japan) were placed along a straight horizontal line and separated by 25 mm and 10 mm, respectively. A simple soundproof enclosure composed of a cardboard box with sound-absorbing material was placed over the setup to reduce interference from external noise.

As shown in Fig 6(A), a sine-wave signal with an amplitude of 0.1 V, an offset of 0.05 V, and a frequency ranging from 1 to 10 kHz was output from a lock-in amplifier (MFLI 500k, Zurich Instruments, Switzerland). The output signal was amplified by a headphone amplifier (AT—HA2, Audio-Technica, Japan) and then output to the speaker. The microphone was connected to a sensor amplifier (SR—2210, Ono Sokki Co., Ltd.), which amplified the signal at a factor of +20 dB. The signal from the sensor amplifier was input to the lock-in amplifier.

The experimental results are plotted in Fig 6(B). The notch frequency was approximately 5.7 kHz in each HR array plate. At each notch frequency, the sound insulation effect was approximately 12 dB for the single plate approximately 42 dB and 48 dB for the two plates installed in the same and opposite directions, respectively. All the gains decreased sharply only around the notch frequencies, confirming that the HR array plates are effective as acoustic notch filters.

The validity and the error of the measurement are evaluated below. Fig 1(C) in S1 Appendix shows the input voltage of the lock-in amplifier *V*_in_ for the measurement results with and without HR array plates. From each *V*_in_, Gain(*F*/*F*_0_) was calculated (Fig 6(B)) relative to *V*_in_ with no plate, where *F* and *F*_0_ denote the transfer functions between the speaker and the microphone with and without plates, respectively. Here, we consider the S/N ratio at the notch frequency where the magnitude of the signal is the smallest, i.e., the maximum error. The measurement error is assumed to be due mainly to the microphone. The input voltage of the lock-in amplifier was -78 dBV at the notch frequency, as shown in Fig 1(C) in S1 Appendix, and the microphone output voltage was calculated as 12.6 μV. As the microphone has a self-noise level of 3.3 μV, and the S/N ratio was calculated to be 3.82. The measurement error was estimated to be ±2.3 dB at the notch frequency, indicating that the measurements are sufficiently reliable.

### Experiment 2: Sound insulation effect of the earmuff device

To evaluate the sound insulation effect of the HR array earmuff when actually worn on the head, we conducted a sound experiment with a dummy head containing a microphone, as shown in Fig 6(C). A hole was positioned at the right ear of the dummy head, and a small microphone was installed inside. Sound was applied from a speaker placed just beside the dummy head microphone in the range of 1–10 kHz. The gain of the device was calculated by taking the difference from the measurement without the device. We experimentally compared the results with three HR array plates configurations: (i) a single plate, (ii) two plates (opposite direction), and (iii) three plates ((ii) + HR array (C) plate).

The experimental procedure is described below. A loudspeaker (FT28D, FOSTEX, Japan) was placed at a vertical distance of 200 mm from the ear canal of the dummy head containing a microphone. The setup was covered with a simple soundproof enclosure to reduce noise. The microphone within the dummy head was connected to a sensor amplifier (SR-2210, Ono Sokki Co., Ltd.), amplified at +20 dB, and then input to a lock-in amplifier. The speaker was connected to a power amplifier (AP20d, FOSTEX), and the power amplifier was connected to the output terminal of the lock-in amplifier. The output signal of the lock-in amplifier was a sine wave with an amplitude of 0.1 V and an offset of 0.05 V. The frequency step was 10 Hz in the range from 1 to 10 kHz.

The dummy head microphone was deployed as follows (Fig 1(D) in S1 Appendix). A hole was casually drilled through the right ear of the dummy head, and a cylinder (fabricated with a 3D printer) with an outer diameter of 21 mm, an inner diameter of 17 mm, and a length of 29 mm was inserted. The inside of the cylinder was filled with a 5 mm thick sponge (KTHU-3015, Hikari, Inc., Japan), which was rolled into a cylindrical shape. The inner diameter of the cylinder filled in with the sponge was 7 mm, which corresponds to the diameter of the average human ear canal. An ultrasmall microphone (MB-2200M10, Ono Sokki Co., Ltd.) was placed at the inner exit of the dummy head, and a lid was placed over the exit to prevent sound inside the dummy head from being recorded by the microphone. The length from the outer exit of the dummy head to the sound hole (sensing point) of the microphone was 25.7 mm, which is also the average length of the human ear canal.

In the experiment, the measurements varied due to slight misalignment of the device when it was attached to the dummy head microphone. Therefore, each experiment was conducted twice, and the average value of the voltage was calculated. Then, Gain(*F*/*F*_0_) was calculated as in Experiment 1 relative to the measurement value without the device.

The experimental results are shown in Fig 6(D). The gains were drastically reduced around the notch frequency (5.7 kHz). Increasing the number of HR array plates improved the sound insulation effect: 27 dB with the single plate, 32 dB with the two plates, and 40 dB with the three plates. In addition, the width of the dip increased with the number of HR array plates. Accordingly, the performance of the acoustic notch filtering device was experimentally confirmed.

Next, we investigated the sound insulation effect of the HR array earmuff when the speaker was placed in front of a dummy head microphone. Experiments were conducted with HR array earmuffs that had two and three plates. A comparison between the placement of the speakers to the side and front of the dummy head microphone is shown in Fig 3 in S1 Appendix. Although a slight decrease in the sound insulation effect is observed due to the horizontal sound wave incidence to the HR array plates when the loudspeaker is placed in the front, they work well enough as notch filtering earmuffs in both conditions.

Furthermore, we compared our proposed device with conventional PNC and ANC earmuffs. The experimental results of the PNC earmuffs (PELTOR X1 Earmuffs X1A, 3M Co., USA) and ANC headphones (WH-1000XM3, Sony Corp., Japan) are shown in Figs 4 and 5 in S1 Appendix. The PNC provided approximately -20~-40 dB of sound insulation in the range of 1–10 kHz. The ANC worked well below 800 Hz but rarely worked above that frequency. On the other hand, the proposed device provided 40 dB of sound insulation around the notch frequency (5.7 kHz) where ANC did not work. The comparison suggests that the proposed device realizes selective sound insulation for high-frequency sounds.

## Discussion

We compared the frequency responses of the HR array plates obtained in Simulation 1 and Experiment 1. Although there are some subtle increases and decreases in the spectra of all the experimental results compared to the simulated results, the overall trends of the two frequency responses are quite similar to each other. The reason for the observed increases and decreases in the experimental results is believed to be due to the influence of echoes from the surrounding walls and experimental equipment. However, in the simulations, all the surrounding boundary surfaces are ideally open, resulting in clean spectra. For both frequency responses, the gain is approximately zero or slightly greater than zero at frequencies lower than 5 kHz and drops sharply over 5 kHz. Above the notch frequency, in the simulation, the gain rises sharply again and then stops rising and remains at approximately -10 dB until reaching 8 kHz. However, in the experiment, the gain rises gradually with some small dips until 8 kHz. For both spectra, the gain remains at approximately 0 dB above 8 kHz until 10 kHz, but there is a slight increase of approximately 5 dB at 4–5 kHz. This is thought to be due to a phase match between the sound waves passing through the hole and those emitted by the HR, causing constructive interference.

The results differ according to the composition of the HR array plates. In both the simulations and the experiments, having two plates increases the depth and width of the dip around the notch frequency compared to the single plate. In addition, considering the relative direction of the two plates, the dip is slightly enlarged when the plates are installed in the opposite direction compared to when they are installed in the same direction. Here, we focus on the difference in the sound insulation effect between the two HR array plates. An enlarged graph is shown in the bottom-right corner of Fig 3(C) for the ranges of 5.5–6.5 kHz and -50~10 dB. Plot (i’), in which the gain of (i) is doubled by a simple calculation and drawn as a yellow dotted line, corresponds to the gain when the two plates are positioned far enough apart. The gain of (iii) is equivalent to that of (i’) around the notch frequency. However, the gain of (ii) is approximately 10 dB higher than that of (i’). This is thought to be because the pressure gradient generated on the surface between the two plates affects the sound insulation since the two plates are placed closely together. In the plots of Fig 3(E) at *x* = 8 mm, one hole on the measurement point side (+*x* side) of the HR array plate is magnified. In Fig 3(E-ii), the pressure difference between the inside of each HR and the centre of the hole provides a circular pressure gradient in the radial direction at *x* = 8 mm. On the other hand, in Fig 3(E-iii), sound waves entering from the three holes positioned symmetrically around the centre hole generate relatively uniform pressure in the hole cross section. This pressure gradient difference between (ii) and (iii) on the *x* = 8 mm plane is thought to affect the sound insulation. The pressure distributions at *x* = 12 and 16 mm show the attenuation of sound through the HR array plate on the +*x* side. At *x* = 16 mm, plot (e-ii) shows a larger pressure gradient than plot (e-iii). As a result, the sound insulation effect is larger when the plates are layered in the opposite direction.

Additionally, we examine the results of Experiment 2 to evaluate the earmuff performance. The results of Experiment 2 show that the responses oscillate as the frequency changes compared with the results of Experiment 1. This is believed to be the result of internal echoes producing constructive and deconstructive interference at the dummy head microphone. The gain of the three-HR-array-plate configuration (iii) is approximately 10 dB lower than those of (i) and (ii) due to the effect of the HR array (C) plate at 4–5 kHz, which is similar to the result of Simulation 2 (Fig 4(C)).

Furthermore, we discuss the results of applying sound waves from the side and front of the dummy head microphone. The sound insulation effects are slightly decreased due to the horizontal sound wave incidence to the HR array plates when the loudspeaker is placed in front of the dummy head microphone. In the 5.5–6.5 kHz range, the sound insulation effect is reduced by 3.5 dB with two plates and by 2.4 dB with three plates on average. The HR array earmuffs with three plates maintain a relatively high sound insulation effect around the notch frequency when the loudspeaker is placed in front. These results suggest that the three HR arrays provide a higher sound insulation effect in diffuse sound fields.

The HR array (C) plate effect is believed to be properly reflected in the experimental results. The experimental results confirm that the three-HR-array-plate configuration achieved a satisfactory sound insulation performance of 40 dB at the notch frequency, which is sufficiently large compared with conventional earmuffs.

The proposed HR array earmuffs can adjust the working frequency by changing the size of the HR unit or the thickness of the HR array plate, although the adjustability is limited due to the space in the earmuffs. In practice, the resonant frequency can be set to above approximately 1 kHz with the same-sized earmuffs by reducing the number of HR units by half, implementing a single thick HR array plate, and elongating the shape of the HR necks. Furthermore, when the dimensions of the HRs are miniaturized to tune the resonance frequency, the viscous effect becomes negligible. The proposed HR array is applicable in other situations in addition to earmuffs. It is noted that the device must be sufficiently large for the dimensions of the HR element. On the other hand, when the HR array plates are applied to large thin areas, we should consider the possibility that the transmission is affected by the bending waves. The proposed earmuff is equipped with two 108 mm diameter HR array plates, each containing 46 HR units, that act as an effective notch filter.

## Conclusion

In this study, we proposed an earmuff-type acoustic notch filter using HR array plates. The designed HR array consists of regular hexagonal penetration hole units and hexagonal parallelogram HR units with an aperture ratio of 0.483. We designed two types of HR array plates: HR array (A, B) plates with a diameter of 108 mm, the necks of which were connected to the inner side of each hole, and HR array (C) plates with a diameter of 57 mm, the necks of which were connected to the front surface of the plate. The HR array (C) plate was designed to insulate sound waves entering through the gap represented by the spongy ear pads. Thus, these two types of HR array plates effectively insulate sound waves entering both perpendicularly and parallel to the array plates.

First, the frequency responses of the designed HR array plates were simulated by applying sound waves both perpendicularly and parallel to the array plates, confirming that the sound insulation effect was sufficiently around the theoretical resonant frequency. The sound insulation effect was higher for perpendicular waves with two HR array plates than with a single HR array plate. Furthermore, the sound insulation effect was improved when the two plates were installed in opposite directions. In this case, the sound insulation effect obtained for the two HR array plates installed in opposite directions was 48 dB but was only 36 dB for the plates oriented in the same direction. When sound waves were applied parallel to the plates, the HR array (C) plate improved the sound insulation effect around the centre of the plates. Among the proposed HR array (C) plate models, the one with HR units densely arranged in a donut shape achieved the highest sound insulation effect, reaching 70 dB. Finally, we fabricated an HR array earmuff with 3D printed HR array plates and an aluminium frame. The experimental frequency responses of the HR array plates were similar to the simulated responses. A sound test via a dummy head microphone showed that the HR array earmuff could achieve a sound insulation effect of approximately 40 dB, which is sufficiently large for an earmuff device.

The designed HR array earmuff has an adjustable notch frequency by modifying the dimensions of the HR units and thus allows the sound insulation performance to be tailored. In other words, by layering the HR array plates with different resonant frequencies or by distributively shifting the unit dimensions, the sound insulation bandwidth is adjustable. The concept of the novel HR array plate is expected to be applied to large areas, such as sound barrier walls. Moreover, the high aperture ratio of the HR plate will be useful in situations requiring permeable airflow. Therefore, the proposed HR array plate has the potential to be applied in various fields other than as earmuffs.

## Supporting information

S1 Appendix Supplementary figures and tables. (PDF)
