# Supplementary material for: Acoustic notch filtering earmuff utilizing Helmholtz resonator arrays
Source: PLoS One. 2021 Oct 19;16(10):e0258842. doi: 10.1371/journal.pone.0258842 (PMC8525754; doi:10.1371/journal.pone.0258842)
Supplement: S1 Appendix — (PDF) [file pone.0258842.s001.pdf]

S1 Appendix. Supplementary figures and tables

S1 Table 1. Element size parameters of the finite element mesh.

Maximum element size	8.75 mm
Minimum element size	0.375 mm
Maximum element growth rate	1.35
Resolution of the curvature	0.3
Resolution of the narrow regions	0.85

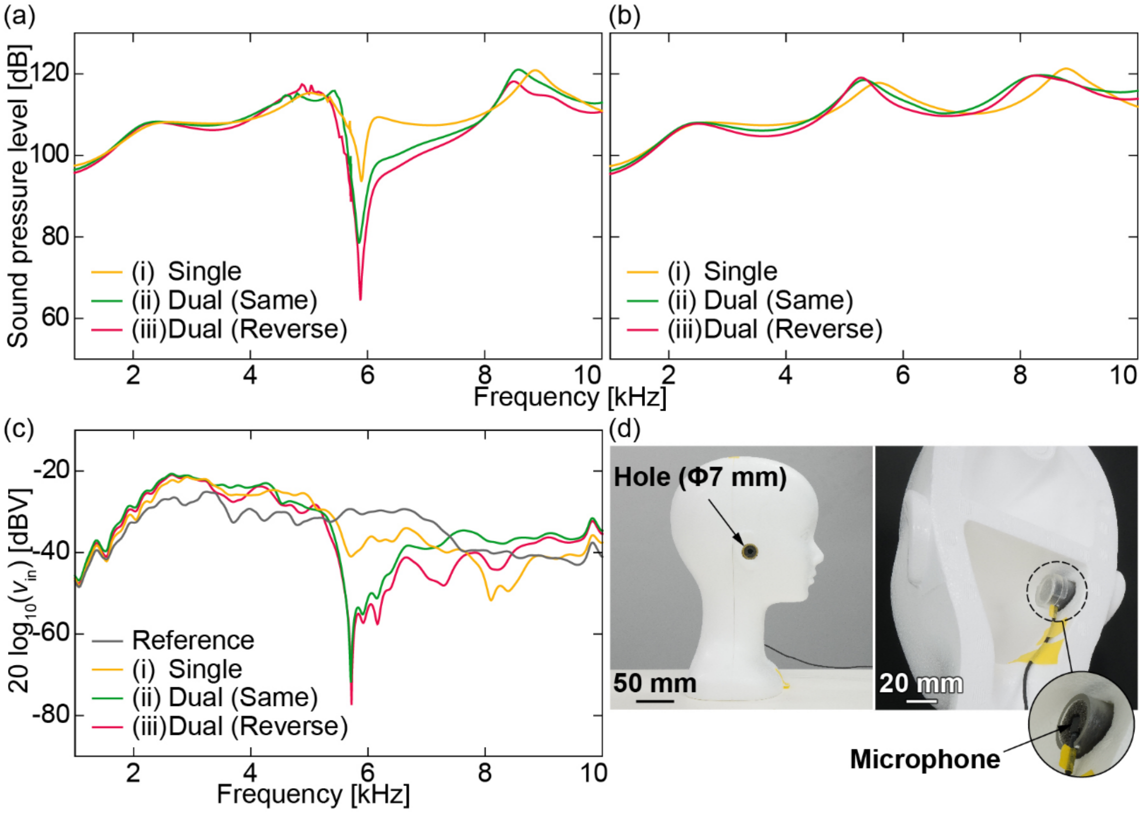

S1 Figure 1. (a) Simulated SPL frequency response. (b) Reference data. The graph shows the SPL frequency response when the HR units are removed from the models. (c) Experimental frequency response of the HR array plate (voltage). Reference data indicate the experimental result without the HR array plates. (d) Dummy head microphone. An ultrasmall microphone was installed inside the dummy head.

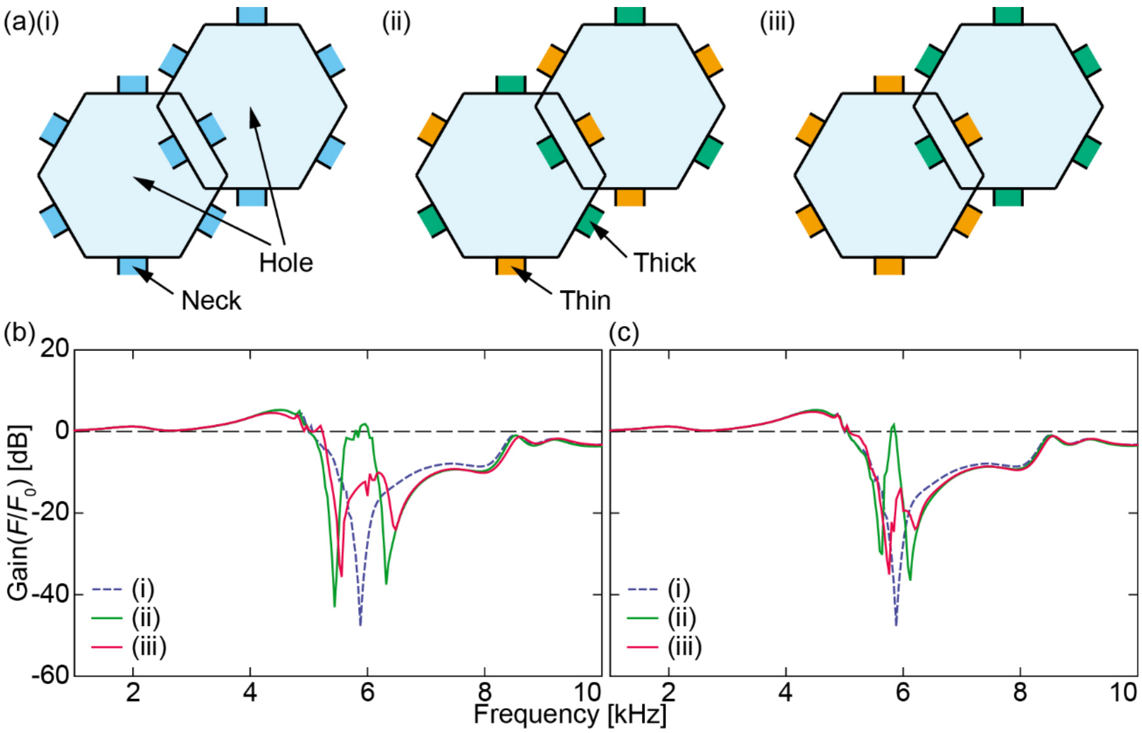

S1 Figure 2. (a) Schematic diagram of the two HR array plates layered in the opposite direction. (i) The radii of all necks are equal. (ii) Neck radii distributed within a single plate. (iii) The radii of all necks in each plate are equal. Each plate has different neck radii. (b) Simulated frequency responses of the three models of (i), (ii), and (iii). In (ii) and (iii), the radii of the necks are shifted by a factor of 1.1. (c) Shift factor is 1.05.

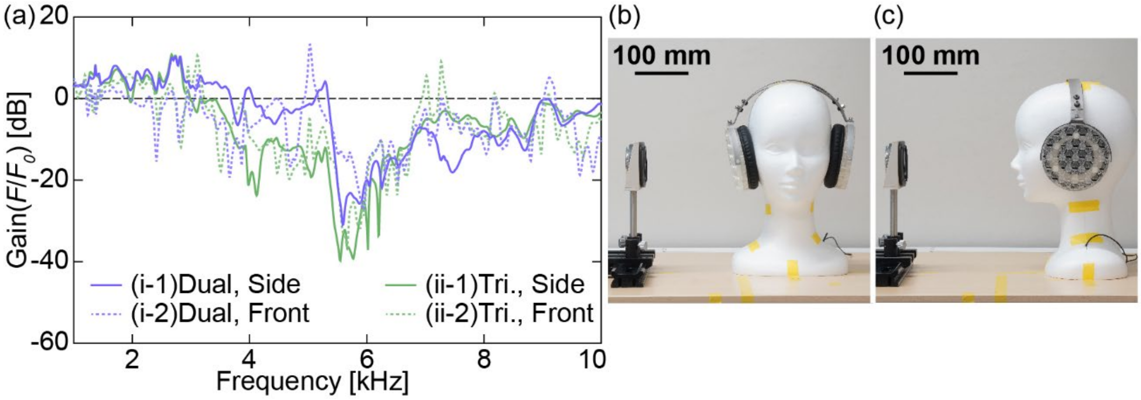

S1 Figure 3. (a) Experimental frequency responses when the speaker is placed to the side and front of the dummy head microphone. Experiments were conducted with HR array earmuffs that had two and three plates. Experimental setup with a speaker to the (b) side and (c) front of the dummy head microphone.

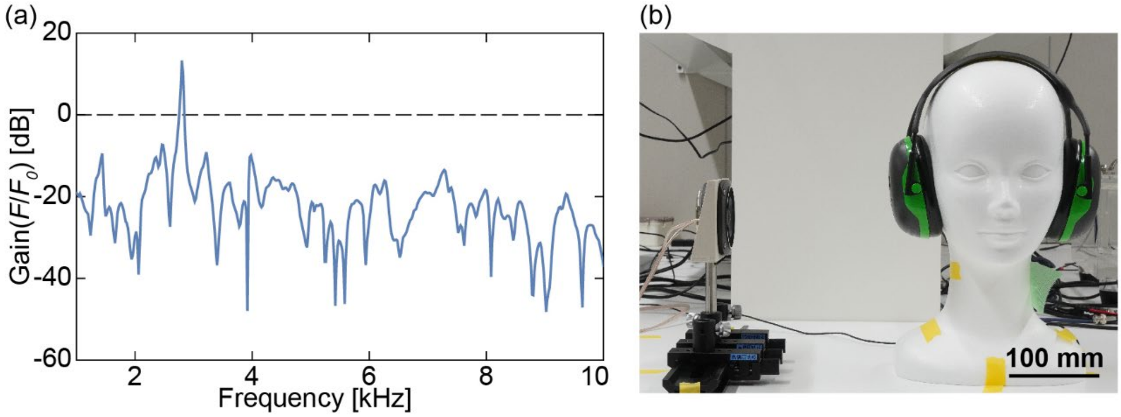

S1 Figure 4. (a) Experimental noise insulation performance of the PNC earmuff (PELTOR X1 Earmuffs X1A, 3M Co.) in the frequency range of 1-10 kHz. (b) Experimental setup. A speaker was placed to the side of the dummy head microphone.

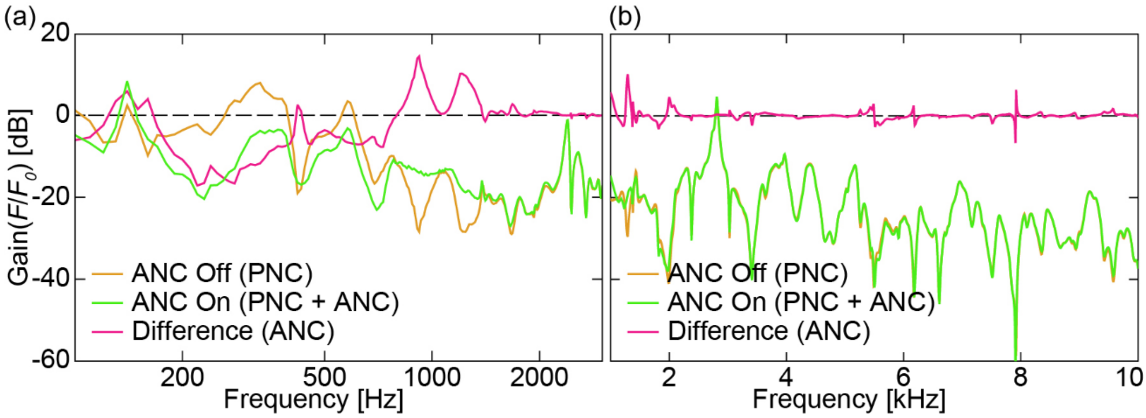

S1 Figure 5. We experimented with the noise insulation performance of ANC headphones (WH-1000XM3, Sony Corp.) in the frequency range of (b) 100-3000 Hz and (c) 1-10 kHz; where we used a woofer (FW208N, FOSTEX) and a tweeter (FT28D, FOSTEX) as the sound sources, respectively. Measurements were made under two conditions, one with ANC turned off and the other with ANC turned on, and the difference (the muffling effect of ANC) was calculated.
